# Beyond the Usual Suspects: Hepatitis E Virus and Its Implications in Hepatocellular Carcinoma

**DOI:** 10.3390/cancers13225867

**Published:** 2021-11-22

**Authors:** Mara Klöhn, Jil Alexandra Schrader, Yannick Brüggemann, Daniel Todt, Eike Steinmann

**Affiliations:** 1 Department of Molecular and Medical Virology, Ruhr-Universität Bochum, 44801 Bochum, Germany; Mara.Kloehn@ruhr-uni-bochum.de (M.K.); Jil.Schrader@ruhr-uni-bochum.de (J.A.S.); Yannick.Brueggemann@ruhr-uni-bochum.de (Y.B.); Daniel.Todt@ruhr-uni-bochum.de (D.T.); 2 European Virus Bioinformatics Center (EVBC), 07743 Jena, Germany; 3 German Centre for Infection Research (DZIF), External Partner Site, 44801 Bochum, Germany

**Keywords:** hepatitis E virus, hepatocellular carcinoma, chronic infection, carcinogenesis, oncovirus

## Abstract

**Simple Summary:**

Cancer is a major threat to global health, accounting worldwide for nearly 10 million fatalities in 2020. Importantly, carcinogenesis caused by so-called oncoviruses accounts for approximately 10% of the global cancer burden. Specifically, hepatitis B virus, hepatitis C virus and to a lesser extent hepatitis D virus infection have been recognized to be mainly responsible for the occurrence of hepatocellular carcinoma (HCC). Recent studies have further drawn attention to a long-neglected hepatotropic virus, namely hepatitis E virus (HEV), in the context of HCC. Here, we summarize current epidemiological, clinical and experimental studies to unravel a putative link between HEV and HCC and provide an outlook for future scientific efforts in HEV-related HCC research.

**Abstract:**

Hepatitis E virus infections are the leading cause of viral hepatitis in humans, contributing to an estimated 3.3 million symptomatic cases and almost 44,000 deaths annually. Recently, HEV infections have been found to result in chronic liver infection and cirrhosis in severely immunocompromised patients, suggesting the possibility of HEV-induced hepatocarcinogenesis. While HEV-associated formation of HCC has rarely been reported, the expansion of HEV’s clinical spectrum and the increasing evidence of chronic HEV infections raise questions about the connection between HEV and HCC. The present review summarizes current clinical evidence of the relationship between HEV and HCC and discusses mechanisms of virus-induced HCC development with regard to HEV pathogenesis. We further elucidate why the development of HEV-induced hepatocellular carcinoma has so rarely been observed and provide an outlook on possible experimental set-ups to study the relationship between HEV and HCC formation.

## 1. Introduction

### 1.1. Hepatocellular Carcinoma

Hepatocellular carcinoma (HCC) is the most common primary liver cancer, accounting for about 75–85% of all primary liver malignancies. With about 830,000 deaths in 2020, HCC remains the third-leading cause of cancer-related mortality globally [1]. Risk factors contributing to the development of HCC generally include high alcohol consumption, obesity, hemochromatosis, dietary aflatoxin exposure, type 2 diabetes, smoking, sex (male) and older age (50+) [2,3]. However, these may substantially vary by region. Most importantly, cirrhosis as a consequence of viral infection with hepatitis B virus (HBV) and/or hepatitis C virus (HCV) has been established as a major risk factor for HCC worldwide. Indeed, 78% of all HCC cases are attributable to HBV (53%) or HCV (25%) infection alone [4]. Current research also suggests hepatitis D virus (HDV) plays a crucial role in HCC formation [5,6].

Intriguingly, several epidemiological studies and a recent case report by Borentain et al. suggest the involvement of hepatitis E virus (HEV) in hepatocarcinogenesis [7]. HEV, a hepatotropic RNA virus, causes acute and chronic infection in the human host. This capacity—among others—certainly makes HEV a potential oncogenic agent involved in cancer formation.

Hence, in this review, we summarize and discuss current epidemiological and clinical studies on HCC and HEV infection and potential molecular mechanisms of HEV-driven hepatocarcinogenesis. Additionally, we give a broad outlook on future clinical, epidemiological and experimental studies to elucidate a putative association of HEV with HCC development.

### 1.2. Hepatitis E Virus—An Emerging Zoonotic Pathogen

HEV is an icosahedral quasi-enveloped, positive-sense, linear, single-stranded (+ss) RNA virus [8,9] of the genus *Orthohepeviridae*. Although HEV primarily infects and replicates in hepatocytes, negative-sense RNA intermediates have been detected in cells of the small intestine [10], colon [11] and neuronal system [12,13,14], suggesting extra-hepatic HEV replication. The species *Orthohepevirus A* is subclassified into eight different genotypes (GT), of which GT1–4 are responsible for the majority of infections in humans [15].

The encapsulated 7.2 kb genome of HEV resembles eukaryotic mRNA with a 5′ 7-methylguanylate cap and a 3′ poly(A)tail, and consists of three major open reading frames (ORFs), viz. ORF1-3 [8,16]: ORF1, the best characterized HEV viral protein, encodes a nonstructural polyprotein (1693 amino acids (aa)) harboring a methyltransferase macro domain (MeT), Y-domain, putative papain-like cysteine protease (PCP), hypervariable region (HVR), X-domain, RNA helicase (Hel) and RNA-dependent RNA polymerase (RdRp) [17]. While ORF1 is pivotal for viral RNA replication, ORF2 encodes the viral capsid protein (660 aa) and is involved in virion assembly, interaction with host cells and immunogenicity [18]. ORF3 overlaps with ORF2 and is translated from a subgenomic RNA into a protein of 113–115 aa [19,20]. The ORF3 protein is believed to be a virally encoded ion channel (viroporin) critical for HEV morphogenesis and infectious particle secretion [21,22].

So far, the viral life cycle of HEV is largely undefined. However, HEV is considered to be a quasi-enveloped virus, with the non-enveloped virus shedding into the bile and feces and with enveloped virus found in the blood of HEV-infected individuals [23,24].

**Figure 1:**
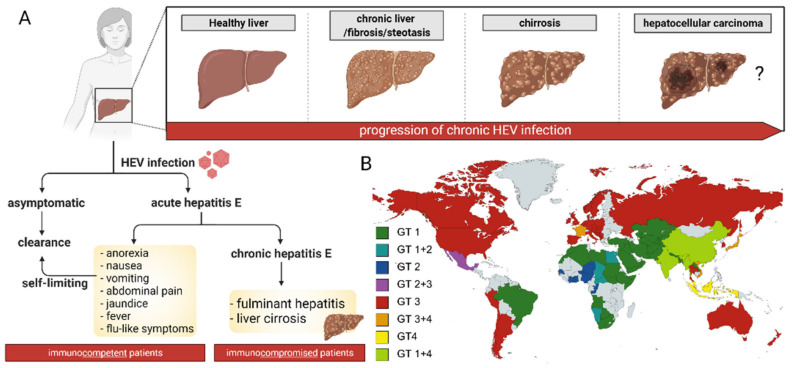
HEV clinical pathology and genotype distribution. (**A**) Progression of chronic HEV infection and clinical course of infection of immunocompetent and immunocompromised patients. (**B**) Geographical distribution of HEV GT1-4 infection. Different colors on the map indicate different HEV genotype (GT1-4) distributions across the globe based on Pallerla et al. [28]. Image was created using BioRender (**A**) and MapChart (**B**).

With an estimated 20 million enterically transmitted HEV infections occurring each year, causing around 44,000–70,000 deaths annually, HEV is a leading cause of acute viral hepatitis [25,26]. A recent meta-analysis of the global HEV epidemiology estimated a worldwide anti-HEV IgG seroprevalence of 12.47% with the highest anti-HEV IgG seropositivity rate in Africa (21.76%), followed by Asia (15.80%) > Europe (9.31%) > North America (8.05%) > South America (7.28%) and Oceania (5.99%) [27,28]. Although HEV infections are present worldwide, there are considerable differences in the distribution, routes of transmission and host reservoir of different genotypes (Figure 1B). GT1 and 2 are limited to infecting human hosts and are endemic in the developing world including Asia, Africa and Latin-America. Infections with GT1 and 2 are mainly transmitted fecal-orally via contaminated drinking water. In contrast, GT3 and 4 are zoonotic and thus found in multiple hosts including humans, swine, wild boar and deer. GT3 and 4 infections are most prevalent in industrialized countries such as Europe or America and are mainly transmitted via undercooked meat or blood transfusions [29].

The current treatment, as recommended by the European Association Study of the Liver (EASL), is based on the administration of the nucleoside analog ribavirin (RBV) and pegylated interferon-α if the reduction of the immunosuppressants does not eliminate the virus infection [30,31]. However, due to contraindications of pegylated interferon-α and the teratogenic nature of ribavirin [32], novel treatment options are urgently needed, and even more so because vaccinations are currently only available in China for GT1 infections [33].

## 2. Clinical Evidence and Epidemiological Studies

Previous studies on hepatotropic viruses including HBV, HCV and HDV have frequently shown the significance of viral infection in HCC development [6,34,35]. Whether HEV has a similar function in hepatocarcinogenesis remains controversial, but several epidemiological and clinical studies imply a potential role of HEV in the HCC pathogenesis.

### 2.1. Acute HEV Infection and Liver Damage—Potential Risk Factor for HCC?

Usually, HEV manifests itself as an acute, asymptomatic and self-limiting infection in immunocompetent healthy individuals (Figure 1) [36]. Regardless, acute liver failure and fulminant hepatitis as a result of acute HEV infection still contribute to fetal, neonatal and maternal mortality of up to 30% in pregnant women in the third trimester and to death in 0.5–4% of patients [28,37,38]. In addition, acute infections with HEV in already damaged livers have been shown to correlate with rapidly progressing cirrhosis, rapid liver decompensation and liver failure [39,40,41]. In this respect, HEV has been found to cause acute-on-chronic liver failure—i.e., acute deterioration of liver function in individuals with compensated chronic liver disease (CLD)—often resulting in an aggravated disease outcome or even death in CLD patients [41,42]. Interestingly, several studies further indicate an indirect link between acute HEV infections and increased HCC incidence. Recently, a nationwide US-American study including >30,000 participants (approx. 6.1% HEV IgG-positive individuals) revealed an interrelation between HEV infections assessed by HEV IgG and a significantly increased likelihood of fibrosis (Table 1) [43].

Likewise, a study investigating 200 HIV-positive Nepalese individuals found a higher fibrosis score in HEV IgG-positive individuals [52]. Correspondingly, in a Spanish cohort including 238 HIV-positive patients, a higher seroprevalence of HEV was found in cirrhosis patients (23%) vs. patients without cirrhosis (6%) [51]. Since fibrosis is a main risk factor leading to cirrhosis, and cirrhosis is a main risk factor for and can often lead to HCC, both studies might imply HEV is a risk factor for HCC development [53]. Furthermore, a case-controlled study in eastern China found that the seroprevalence of anti-HEV antibodies was significantly higher in cancer patients (26.0%), especially leukemia (32.3%) and liver cancer (31.1%) patients, compared to a control group (13%) [46]. However, one has to consider that the Spanish HIV-positive and the eastern Chinese cancer patient cohorts might have been generally more likely to develop more severe HEV infections due to blood transfusions, cancer chemotherapy or immunosuppressive drugs. Most importantly, the US-American and Chinese studies did not consider the HBV and HCV status of participants. While all these studies might demonstrate an association between HEV infection and HCC, an epidemiological study from Croatia on the burden of HEV infection in chronic liver disease concluded differently: in a cohort of 438 CLD patients, they did not find a relationship between HEV seropositivity and HCC incidence [44].

Furthermore, several studies have demonstrated an association between HEV infection and HCC in HBV- or HCV-positive patients. When comparing patients with hepatitis B- and C-related HCC to those with CLD, a Cameroonian case-control study with 67 patients, conducted by Amougou et al., discovered that hepatitis B- and C-related HCC patients had a higher prevalence of having previously been infected with HEV (41.8%) than in controls with CLD but not HCC (14.9%) [48]. They also discovered that HEV-infected hepatitis B- and C-related HCC patients displayed more profound liver deterioration, assessed via elevated levels of liver enzymes and past or recent HEV-seroconversion, adding to the finding that HEV infections might decrease liver capacity and could promote HCC. Even though differentiation between HEV genotypes and the occurrence of HCC as well as data from a healthy control group were missing, the correlation of HCC with a higher seroprevalence of HEV was clear. Moreover, in 2020, a Taiwanese study linked an increased long-term risk of cirrhosis and HCC and accelerated disease progression with HEV superinfection in HBeAg-negative chronically HBV-infected subjects. However, conclusions have to be drawn carefully here since the data give adverse results for the overall chronic HBV-infected cohort [49]. Additionally, in Ghana (West Africa), Owusu et al. conducted a cross-sectional study of 155 patients with jaundice, 32 of whom were diagnosed with HCC [45]. Interestingly, among these HCC patients, nine study participants were found to be coinfected with HBV and HEV, whereas only two patients presented with single HEV infection, indicating either the important role HEV might play in causing HCC, or as the author discusses, the higher susceptibility of HBV-infected patients to HEV infections. A contradictory study, however, did not find an increased risk of HCC after HEV superinfection in chronically HBV-infected patients, in a cohort consisting of 474 HCC patients and 586 controls [47].

Considering all epidemiological data g, it appears that acute HEV infections might be a contributing factor facilitating the progression of chronic liver disease toward HCC in liver tissue already primed for hepatocellular carcinogenesis (e.g., by HBV/HCV infection). It also seems HEV infections increase the risk of fibrosis, cirrhosis and thus eventually, HCC. However, the effect appears to be diminutive, contradictory data exist and conclusions must be drawn carefully since further studies are lacking.

### 2.2. Chronic HEV Infection Might Aggravate HCC Occurrence in Cirrhotic Patients

Similar to HCV and HBV infection, HEV GT3, and in rare occasions GT4, have been known to cause persistent infection in immunosuppressed patients like liver and kidney (-pancreas) organ recipients [54,55,56], in patients suffering from non-Hodgkin lymphoma [57] or individuals infected with human immunodeficiency virus (HIV) [58,59,60,61]. In these patients, long-term clinical manifestations of persistent HEV infections ranged from chronic inflammation to rapid liver fibrosis, cirrhosis and death due to decompensated cirrhosis [39,50,54]. Since chronic inflammation in the liver is associated with an increased likelihood of cirrhosis and thus also HCC [53], the question of whether chronic HEV infections could increase the risk of the development of HCC, therefore, has to be addressed separately.

A study of 56 chronically HEV-infected transplant recipients not only described HEV as the causative agent of death due to decompensated cirrhosis, but also reported a liver-transplant recipient coinfected with HBV with a relapse of HCC [50]. Similarly, another case report described the relapse and rapid acceleration of HCC in a Chinese HBV-coinfected liver cirrhosis patient chronically infected with HEV [62]. Even though the HEV RNA levels and the genotype were not determined, both studies suggest the important role of chronic HEV infections as a promoting factor accelerating HCC in patients with further etiologies for liver disease, although only in very rare cases.

Nonetheless, whether HEV alone and without other etiologies has the ability to induce HCC remains to be elucidated. Strikingly, in 2018, Borentain et al. [7] described a case of HCC that was most likely induced by chronic infection with HEV. A 68-year old male farmer, who had previously been diagnosed with follicular lymphoma (a type of non-Hodgkin lymphoma affecting the lymphatic system), but was considered cured, had suffered from chronic HEV lasting for eight years before the discovery of HCC. Importantly, other major etiologies for cirrhosis and HCC including HBV, HCV infection, autoantibodies, consumption of alcohol and metabolic syndrome were excluded [7]. While previously undiscovered hepato-damaging factors cannot be completely dismissed, the authors concluded that HEV-induced HCC on the basis of chronic inflammation, fibrosis and cirrhosis of the liver provided a likely explanation for their observations. This case was the first ever reported case of HCC induced by chronic inflammation due to a persistent HEV infection.

Taken together, while epidemiological data suggest acute infections of HEV increase the risk of HCC, studies present the possibility that chronic HEV infections could not only promote and accelerate HCC in previously damaged livers but might also be able to cause HCC without other promoting factors of liver diseases. However, the clinical impact appears to be rather minor with so far very few cases reported.

## 3. Putative Molecular Factors Involved in HEV-Mediated HCC—Hallmarks of Cancer

**Figure 2:**
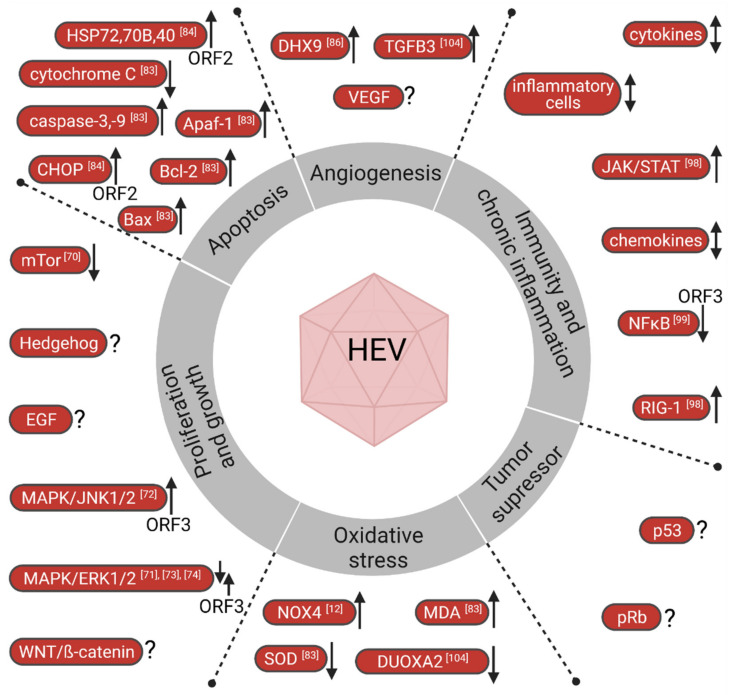
HEV involvement in known cancer pathways and outlook on putative research targets. Upward facing arrows indicate upregulation/activation while downward facing arrows indicate downregulation/inhibition of respective factors. Question marks indicate knowledge gaps of recognized cancer-induced factors, paired arrows facing up and down indicate contradicting data and up-down arrows indicate groups of signal proteins that could be activated and inhibited. Illustration was created with BioRender.com.

In 2011, Douglas Hanahan and Robert A. Weinberg provided a comprehensible framework to organize the complexities of neoplastic disease, known as the “hallmarks of cancer” [63,64]. These hallmarks include sustaining proliferative signaling, evading growth suppressors, resisting cell death, enabling replicative immortality, inducing angiogenesis, activating invasion and metastasis, reprogramming energy metabolism and evading immune destruction [63,64]. Likewise, alteration of cellular signaling pathways, genetic alterations and chronic inflammation are considered to be key processes involved in HCC progression and carcinogenesis. Despite the limitations of requiring a sufficient cell culture system and in vivo models for HEV research, numerous studies have investigated HEV’s involvement and disruption of numerous pathways associated with virus-induced hepatocarcinogenesis (Figure 2).

### 3.1. Deregulation, Corruption and Evasion of Cellular Signaling Pathways by HEV

A major pathogenic mechanism underlying hepatocarcinogenesis is the disruption and inhibition of cell signaling cascades of tumor suppressor proteins and activation and upregulation of growth and proliferation pathways. In particular, the signaling cascades activated by G-protein coupled receptors and receptor tyrosine kinases like EGFR, namely the mitogen-activated protein kinase (MAPK)/ERK (or Ras/Raf/MEK/ERK) and PI3K/AKT/mTOR pathways, have been identified to be involved in the oncogenic process [65]. Interestingly, numerous studies conducted on HBV-, HDV- and HCV-associated HCC have been unraveling how deregulation of these cellular pathways by viral proteins drives hepatocarcinogenesis (i.e., reviewed in [66,67,68]). Interestingly, several studies have identified tumorigenesis pathways to be deregulated by HEV infection.

For one, mammalian target of rapamycin (mTOR), a serine-threonine kinase and master-regulator of the phosphatidylinositol-3 kinase (PI3K)-protein kinase B (PKB)-mTOR signaling cascade, regulates cellular growth, proliferation and cell metabolism and was found to inhibit growth factor signaling and catabolism when activated [69]. Interestingly, administration of rapalogs (mTOR inhibitors) as immunosuppressants is associated with aggravated risk of developing chronic hepatitis E in organ recipients [55]. Additionally, mTOR inhibition was shown to significantly increase viral HEV replication in hepatocyte-like cells infected with full-length HEV virus. Similarly, inhibition of upstream elements of the mTOR signaling cascade PI3K-PKB promotes HEV replication, while simultaneous inhibition of PI3K and mTOR further enhances viral replication [70]. Another study by Gong et al. [71] found that hepatitis E viral infection significantly suppresses the PI3K/AKT/mTOR signaling pathway in human lung carcinoma cells. While the PI3K-AKT-mTOR pathway might be altered during HEV infection, the same study suggested that the Ras/Raf/MEK/ERK signaling cascade, a pathway that was found to contribute to HCC via ERK1/2 activation, is independent of HEV infection [71]. However, to put these findings into perspective, this study conducted experiments in adenocarcinoma human alveolar basal epithelial cells with infection of the GT4 isolated from swine, which might limit the significance of the results regarding human liver cancer. In addition contradicting data exist, suggesting the activation of MAPK/ERK and MAPK/JNK1/2 through ORF3 [72,73,74].

So far, in vitro and in vivo research on pathways known to regulate cancer formation and hepatocyte transformation, including the Hedgehog, WNT/β-catenin, hepatocyte growth factor/c-MET and vascular endothelial growth factor (VEGF) cascades, is yet to be produced to our knowledge. Similarly, current data are missing on the evasion of p53 and retinoblastoma protein (pRb) during HEV virus infection, two major tumor repressors involved in cancer formation and widely recognized master-regulators of oncogenesis [75,76].

Other regulatory molecules involved in cancer formation are microRNAs. These small non-coding RNAs are characterized by their involvement in the maintenance of hepatic differentiation and function, as well as suppression of cell proliferation and apoptosis [77,78,79]. In particular, miR122-5p has been found to stabilize the HCV genome and subsequent enhancement of replication [80]. Notably, a recent in vitro study has shown a similar effect of miR-122-3p on HEV replication [81]. Another microRNA, miR-99a-5p, shown to modulate HBV replication by promoting IGF-1R/PI3K/Akt/mTOR/ULK1 signaling-induced autophagy, has been found to be upregulated in acute and chronic HEV patients [82].

In addition, major apoptosis and necrosis processes and signaling pathways must be altered by virus infection to induce cancer formation. Recently, Yang et al. found increased levels of mitochondrion-mediated apoptosis-regulating proteins Bax and anti-apoptotic protein Bcl-2 in HEV GT4-infected Mongolian gerbils [83]. Similarly, apoptotic protease activating factor 1 (Apaf-1) and active caspase-3 and -9 levels were elevated, while cytochrome C protein levels were reduced in HEV-infected gerbils [83]. Another study found evidence of ORF2-driven evasion from cellular apoptosis. They observed that upregulation of pro-apoptotic gene CHOP did not lead to apoptosis, while concomitant expression of anti-apoptotic heat-shock proteins was upregulated [84].

Other lesser-known proteins involved in angiogenesis, such as the DHX9 protein, have recently been suggested to be involved in various cancers [85]. Interestingly, Paingankar and Arankalle identified DHX9 to be hijacked by HEV to enhance infection efficiency [86]. Finally, frequently utilized cancer-causing modes-of-action, such as the expression of viral oncogenic proteins, that directly cause hepatocarcinogenesis (e.g., HbX protein from HBV [87]) have not been shown to be the case for HEV. Similarly, the integration of viral DNA into the host genome, a mechanism prominently exploited by oncoviruses with a DNA genome (e.g., HBV, HIV), is unlikely for HEV, since HEV is an RNA virus without a DNA intermediate.

### 3.2. Chronic Inflammation—Indirect Process in HEV-Driven HCC Carcinogenesis?

Arguably, chronic inflammation is one of the most fundamental factors promoting anti-tumor immunity and HCC carcinogenesis. Chronic inflammation as a result of persistent viral infection is characterized by ongoing cell death and long-term local infiltration of inflammatory cells (e.g., macrophages, mast cells, neutrophils, T- and B-lymphocytes) [66]. During the active immune response, the cellular microenvironment is highly altered owing to a great abundance of highly reactive oxygen and nitrate species (ROS and RNS), cytokines, chemokines and growth factors [88]. NK-cells and T-cells releasing a great amount of ROS and RNS lead to increased oxidative stress, which in turn, can cause DNA damage and mutations via DNA modifications or protein damage to DNA repair enzymes and caspases inducing oncogenic mutations and epigenetic modifications (reviewed in [89,90]). Subsequently, increased cellular proliferation is favored due to the production of myriad cytokines, chemokines and growth factors, and further due to the peroxidation of lipids and activation of the arachidonic acid cascade [91]. Ultimately, these two circumstances may have a synergistic effect that causes cells initiated for cancer to continue to proliferate in a microenvironment full of inflammatory cells and growth/survival factors that support their growth.

So far, comprehensive in vitro and in vivo studies have explored the mechanisms of inflammation and host immune response in acute hepatitis E patients [92,93,94,95,96,97,98,99]. For instance, immunohistochemistry staining of liver biopsy samples of patients diagnosed with HEV-related acute liver failure showed an increased infiltration of cytotoxic T-cells (CD3, CD8), natural killer cells (NK-cells; CD56) and helper T-cells (CD4) compared to liver biopsies of healthy patients [95]. Similarly, Agrawal et al. demonstrated CD3 + T-cell and cytotoxic (CD8+) T-cell infiltration into the liver of acute fulminant hepatitis E patients, indicating that CD8+ lymphocytes play a role in HEV-induced liver injury [97]. Regardless, these studies do not explore the possibility of persistent recruitment of tumor-promoting immune inflammatory cells in sites of chronic inflammation. Histology results of one study by Galante et al. of biopsy specimens of three patients chronically infected with HEV were found to show signs of mild-to-moderate inflammation [100]. On the contrary, in vitro stimulation of peripheral blood mononuclear cells (PBMCs) from chronically HEV-infected patients with HEV-overlapping peptide pools spanning all proteins encoded by HEV-ORF2 and HEV-ORF3 found that proliferation as well as cytokine production of CD4+ and CD8+ T cell responses were absent in chronically infected patients [94]. Studies such as these—or rather, the lack thereof—make it increasingly apparent that further studies are warranted to determine whether tumor-promoting inflammatory cells persist in chronically infected HEV patients.

Cytokines and chemokines are released in response to cellular stresses including infection, inflammation and injury. Failure to resolve these stresses may lead to excessive infiltration of immune cells and provoke persistent cytokine production [101]. Current studies have demonstrated the mobilization of (memory) γδ T cells and production of anti-inflammatory interleukin-10 in acute HEV-infected patients [102]. Additionally, Wu et al. observed a decrease of pro-inflammatory interferon γ production accompanied by an increased frequency of anti-inflammatory Th2 cytokines in acute HEV cases [103]. Regardless, whether cytokine and chemokine excretion can persist in HEV-infected individuals, causing chronic inflammation of liver tissues, remains subject to future research.

**Figure 3:**
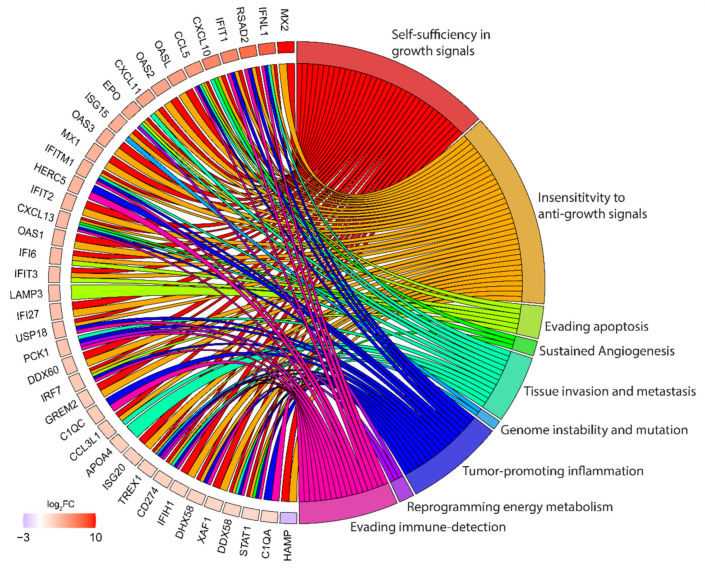
GO cluster plot showing a chord dendrogram of the clustering of the expression spectrum of significantly deregulated genes. Gene ontology identifiers were retrieved from Chen et al. [106] and transcriptome data were provided by Todt et al. [105]. Only transcripts that were deregulated at least four-fold were plotted. Color code represents the logarithmic fold-change (log_2_FC) of mRNA expression in HEVcc-infected PHHs compared to uninfected cells at 48 h post-infection with HEVcc. Gene identifiers for transcripts with absolute log_2_FC ≥ 2, RPKM ≥ 0.5 were used as the input for identification of significantly enriched GO categories.

RNA sequencing (RNA-seq) data from Huh-7 cells transfected with HEV GT1 replicon identifies upregulation of a set of pro-inflammatory agents [104]. For instance, levels of platelet/endothelial cell adhesion molecule 1 (PECAM1) were upregulated, suggesting alterations of leukocyte trans-endothelial migration in HEV-transfected cells. Similarly, CCL27 and selectin P ligand (SELPLG), two pro-inflammatory agents involved in triggering T-cell mediated inflammation and leukocyte trafficking, were upregulated in HEV-transfected cells. Yet another example involved the upregulation of transforming growth factor-ß (TGF-ß) subtype TGFB3, a key player in chronic liver disease [104]. In addition, RNA-seq data of cell-culture-derived HEV (HEVcc)-infected primary human hepatocytes (PHHs) identified deregulation of expressed genes [105]. We have used these sequencing datasets to link changes in gene expression during HEV virus infection with the gene ontology for cancer (retrieved from Chen et al. [106]) (Figure 3). Similar to Jagya et al., numerous genes that are integrated in the hallmarks of cancer are deregulated. For instance, numerous transcripts found to be involved in “self-sufficiency in growth signals” and “insensitivity of anti-growth signals” (e.g., MX2, OASL, OAS2, MX1, OAS3, IFIT2, GREM2) are upregulated (indicated as red squares) in HEV-infected PHHs. Others are upregulated and involved in more than two hallmarks of cancer (e.g., CXCL10, CCL5, EPO, CXCL13, TREX1). Also, HAMP, which is involved in “self-sufficiency in growth signal” and “insensitivity to anti-growth signals,” is downregulated in HEVcc-infected PHHs.

In contrast to these data, Crouchet et al., who developed an in vitro system that recapitulates clinical prognostic liver signatures (PLS) that are predictive of long-term liver disease progression toward HCC, found induction of these signatures to be absent, suggesting that HEV might not be associated with liver cancer [107].

Apart from cytokines and chemokines, ROS and RNS play an equally important role in facilitating neoplastic progression. A study by Bhatnagar et al. found oxidative stress to be present during pregnancies of HEV-infected women by observing reduced glutathione (GSH) levels in pregnant women infected with HEV compared to healthy pregnant controls [108]. Another recent study from northeast India identified increased levels of homocysteine [109], an amino acid found to induce production of reactive oxygen species, in pregnant women suffering from HEV infection [110]. Additionally, a study in primary human brain microvascular endothelial cells (HBMVCs) infected with swine-derived HEV virus suggested ROS accumulation through upregulated expression of NADPH oxidase 4 (NOX4) [12]. On the contrary, mRNA levels of another NADPH oxidase, namely dual oxidase (DUOXA2), were downregulated in HBV/HEV replicon coinfected Huh-7 cells [104]. Collectively, observations like these indicate that the formation of ROS and RNS might contribute significantly to HEV pathogenesis; however, it is presently unresolved whether ROS and RNS are produced in the HEV-infected liver and whether they can contribute to development of hepatocellular carcinoma in HEV-infected individuals.

## 4. Future Perspectives

Currently, scientific evidence for HEV-related hepatocarcinogenesis is based on very few epidemiological and biological data. However, several studies discuss HEV as a possible risk factor for HCC acceleration, and chronic HEV infections may lead in untreated cases to cancer progression possibly via chronic inflammation and decrease in liver capacity in patients primed for liver damage. Despite limited data, HEV may act as an indirect carcinogen, although contributing seemingly only marginally. Additionally, only one clinical case of HEV-related HCC has been observed to date [7]. One might interpret this lack of further studies as a sign that HEV is indeed incapable of inducing carcinogenesis, and more so because the clinical endpoint of untreated chronically infected individuals is often reached quite rapidly (in immunocompromised solid-organ transplant patients, ~ 10% under-go cirrhosis within two years) [111]. Virus-induced cancers, however, usually appear many years to decades after an initial infection. For instance, other hepatotropic oncoviruses such as HBV or HCV usually lead to HCC 20–30 years post-infection [112]. On the other hand, usually only a small fraction of individuals infected with an oncovirus eventually develop a tumor. Globally, HEV is not routinely tested and overall case numbers of chronic HEV are already low, suggesting that the number of HEV-induced HCC cases might be lower compared to other etiologies, which might explain why HEV-related HCC has rarely been reported so far. Also, for some oncoviruses (e.g., HCV), risks of cancer have been found to persist even after curing the infection with direct-acting antivirals [113]. In this regard, several studies recently observed persistent alterations of the immune system despite early treatment and short duration of viremia [114,115]. Whether HEV might use similar mechanisms to cause HCC remains unexplored and has to be determined in future research.

Furthermore, for decades, a major obstacle in studying virus-host interactions, biomolecular processes of HEV infections and pathology has been the lack of a robust cell culture systems and efficient propagation of infectious HEV particles in vitro. Ultimately, major gaps in the knowledge of HEV’s pathogenesis can be assumed to limit the understanding of HEV’s possible oncogenic character. However, with increasing awareness of HEV infections in Europe and North America, concomitant with extended testing, a possible epidemiological correlation between HEV infections and an increased risk of HCC can be assessed. Moreover, emerging advances in animal and in vitro models for HEV research [24,105] (as discussed below) might be harnessed for HEV-related HCC studies and help decipher putative underlying molecular mechanisms.

Subsequently, given the disparate nature of virus-induced cancers, limited availability of animal models and the inherent complexity of virus–host interactions involved in cancer development and progression [116,117], studying HEV-related hepatocarcinogenesis faces considerable challenges. In this regard, zur Hausen et al. defined criteria (i.e., epidemiological evidence for oncovirus as risk factor for cancer development, detection of oncovirus genome in HCC tumor tissue, determining biological plausibility, finding evidence that cancer formation relies on continuous expression of viral oncoproteins) to determine whether a virus is able to induce cells to become tumorigenic and ultimately malignant [118]. As outlined below, some HEV studies have already met some criteria, while other questions have yet to be addressed:

### 4.1. Epidemiological Investigation of HEV-Associated HCC Risk

In order for a virus to be considered an oncogenic virus, studies must present epidemiological evidence that HEV infections represent a major risk factor for cancer development [118]. As summarized above, most current epidemiological studies are limited to regions in China and sub-Saharan Africa with only few studies in Europe, the rest of Asia, Australia, Oceania and the Americas. Hence, it remains to be determined whether HEV-infected patients from all continents are similarly likely or unlikely to develop HCC on HEV infection. Concurrently, studies on the influences of different genotypes, including differences between GT1 and 2 that only cause acute infections and GT3 and 4 that also cause chronic hepatitis E, are currently missing. Future epidemiological studies should, therefore, focus on large and worldwide patient cohorts investigating the HEV seroprevalence and infection rate in HCC patients vs. matched healthy controls, and thus evaluating HEV as a risk factor for HCC via a retro-perspective approach and taking genotypes and acuteness of infections into account. Furthermore, the interrelation between HEV seroprevalence or infection and risk of liver disease including HCC should be investigated nationwide and worldwide, analyzing all HEV-infected individuals, including follow-up studies after HEV clearance. This could not only give hints of the HCC risk on HEV infection but also further subclinical effects like alterations of the immune system found after curing HCV infections [113,114,115]. A respective analysis of a >30,000-participant cohort, including approx. >1830 HEV IgG-positive individuals, was recently published from the USA, discovering a significant increase in the likelihood of having significant fibrosis [43], but studies from other countries with similarly high case numbers are lacking so far. Additionally, follow-up studies of chronically infected patients should be conducted, investigating long-term effects of chronic HEV-induced inflammation.

### 4.2. Detection of HEV Genome in HCC Tumor Tissue

Apart from comprehensive, large-scale epidemiological studies, future research needs to demonstrate the regular presence and persistence of HEV in tumor biopsies. To our knowledge Borentain et al. were the only group able to demonstrate the presence of HEV RNA in tumor biopsies and healthy tissue of a patient suffering from HCC [7], and thus further surveillance is needed.

Notably, determining the presence of HEV in liver cancer tissue is by no means sufficient enough to determine whether HEV is able to induce hepatocarcinogenesis, but merely provides an indication. Cancer cells could simply be more susceptible to HEV infection. Therefore, studies that clearly demonstrate biological plausibility will be instrumental in determining the relationship between HEV and HCC.

### 4.3. Establishing Biological Causality

To establish biological causality, studies utilizing in vitro tissue cell culture or suitable animal models need to demonstrate HEV’s immortalizing and transforming activity as well as identify key regulators of cell transformation (e.g., viral proteins). In general, transformed cells acquire their tumorigenic features through a multifactorial biological process that integrates many of the “hallmarks of cancer” [63,116].

As a first starting point, studies examining the cell-transforming ability of HCV could be used to study a putative immortalizing activity of HEV. For instance, studies on HCV employed nontumorigenic rat fibroblast (RF) cells to study HCV-encoded proteins involved in the tumorigenic process [119]. Similarly, Li et al. utilized normal human biliary epithelial (hBE) cells to investigate signaling of malignant transformation [120]. Interestingly, Bose et al. observed an extended lifespan (more than 12 weeks) of primary human hepatocytes infected in vitro with cell culture-grown HCV GT1a or 2a [121]. These hepatocytes were also found to express a number of cancer stem-like cell (CSC) markers [122]. Since Todt et al. [105] have successfully demonstrated in vitro infection with HEVcc of PHHs, one could start to investigate a putative cell-transforming activity of HEV in PHHs, similar to Bose et al. [121].

Apart from in vitro systems, animal models are equally if not more important for determining HEV involvement in hepatocarcinogenesis, and more so because HEV-driven HCC might be highly dependent on prolonged inflammation and liver cirrhosis (as is the case for HCV and HBV), which can ideally be studied in vivo.

Despite HEV’s broad host range, most animals available for in vivo experimentation are disadvantageous to study HEV-related HCC due to the inability of certain species to experience chronic disease progression when infected with human pathogenic strains (e.g., rabbits) or the fact that some species can only be infected with genetically distant HEV strains (e.g., chicken, ferrets and rats) [123,124,125,126]. Current state-of-the-art animal models to study HEV pathogenesis and chronic HEV infection consist of human liver chimeric mice [125] and pigs [127,128]. However, the latter only mimics chronic infection through administration of immunosuppressive drugs [127]. In addition, pig models can only be employed as an animal model for GT3 and GT4 infection since they are not susceptible to GT1 and 2 [128]. In contrast, humanized mice might potentially be used to studying HEV-driven HCC development in vivo. For instance, homozygous primary human hepatocyte-engrafted urokinase-type plasminogen activator (uPA) uPA+/+-SCID mice were found to be capable of being infected with HEVcc and HEV of different sources [125]. Moreover, they observed HEV infection to evolve to chronicity (persistent infection of up to 12 weeks). Another group around Allweiss et al. were able to detect persistent viremia in HEV (GT1, GT3)-infected humanized uPA/SCID/beige mice for up to six to seven months, albeit without observing hepatocyte damage or tumor formation [124]. In comparison, 11 of 42 similar human liver chimeric mice (MUP-uPA/SCID/beige) infected with HCV developed tumors four to six months post-infection [129]. The obtained liver tissue of these HCV-infected mice developed HCC. HCC-negative but HCV-infected mice were also used to compare the expression levels of oncoproteins and tumor suppressor proteins from liver tissues. Similarly, utilizing liver tissue samples from HEV-infected humanized mice and prolonging the duration of infection in these animals might give more insights into the possibility of HEV-induced tumorigenesis.

With the advent of next-generation sequencing techniques, understanding and investigating the complexity of molecular pathways and regulators in cancer development is now feasible. For instance, by integrating whole-genome sequencing and transcriptome data, Chen et al. were able to find regulatory genes involved in HCV-HCC development [130]. However, these kinds of studies rely on expression data of liver hepatocellular carcinoma samples infected with HEV (which are rare, as discussed above).

### 4.4. Constitutively Expression of Viral Oncoproteins

Based on zur Hausen’s defined criteria for direct-acting oncogenic viruses, it must be demonstrated that the malignant phenotype depends on the continuous expression of viral oncogenes or on the modification of host-cell genes containing viral sequences [118].

Unlike HBV, HEV is an RNA virus without the ability to integrate into the host’s genomic DNA, and hence, modification of host-cell genes by integrating viral sequences is an impossible mechanism for oncogenesis of HEV. Nevertheless, viruses are known to possess cell-transforming abilities via the interference of viral proteins with host-cell pathways. For example, HCV viral protein NS3 was found to induce cell transformation via interference with wide a range of proliferative signaling pathways (reviewed by Goto et al. [131]). To study putative mechanisms for HEV-related HCC, cell and animal models that are able to constitutively express viral proteins (e.g., transgenic mice, stem cell-derived human hepatocytes), as described above, can be utilized.

## 5. Conclusions

Identifying oncovirus infections as the causative agent of cancer is arguably one of the major achievements in cancer research of the past decades. Recent epidemiological and clinical studies have advanced the possibility of hepatitis E virus infections as a promoting factor for hepatocellular carcinoma, although the clinical significance is considered low. However, whether HEV facilitates neoplastic progression of acutely or chronically infected patients remains poorly understood. Hence, demonstrating cell transformation using in vitro and animal models and oncogenic programs potentially induced by HEV certainly represents an important agenda for future research. Increasing awareness and expanding frequent diagnostic testing of acute and chronic HEV infections, as well as routinely screening chronic HEV patients for HCC, will help elucidate a putative involvement of HEV in HCC development.

## Figures and Tables

**Table 1:** Epidemiological studies and case reports on HEV-associated hepatocellular carcinoma.

Cohort	Region	Year of Publication	Observation Regarding HEV and HCC	Reference
6.1% HEV IgG-positive participants out of >30,000 subjects	USA	2021	HEV IgG-positive individuals have a statistically significant increase in the likelihood of having fibrosis measured as a Fib-4 score > 1.45	[43]
107 cirrhotic patients and 200 healthy controls	India	2007	Higher prevalence of HEV RNA in cirrhotic patients (28%) vs. healthy controls (4.5%), and higher mortality rate at four weeks in HEV-infected cirrhotic patients (43%) vs. HEV RNA-negative cirrhotic patients (22%), showing that HEV infections in cirrhotic patients are associated with rapid liver decompensation and death	[39]
438 CLD patients	Croatia	2020	HEV seropositivity and HCC incidence were not related	[44]
155 jaundiced patients	Ghana	2018	Most of the cases found of HEV were coinfections with HBV with the predominant clinical manifestation being HCC	[45]
950 cancer patients and 950 controls	Shandong, Eastern China	2018	Overall, seroprevalence of HEV IgG and IgM is significantly higher in cancer patients (26.0%), especially in leukemia (32.3%) and liver cancer (31.1%) patients, than in controls (13.0%)	[46]
474 HCC patients and 586 non-cancer patients	Guangzhou, China	2020	HEV infection was not an independent risk factor for HCC but coinfection of HBV and HEV might be positively associated with HCC development	[47]
67 HCC patients (47 HBV- and 20 HCV-related), 67 CLD patients (47 HBV- and 20 HCV-related) and 67 patients with no liver disease	Cameroon	2017	HCC patients have a higher seroprevalence of HEV IgG (41.8%) compared to CLD patients; HEV IgG-positive HCC patients have more profound alterations of circulating liver enzymes compared to HEV IgG-negative HCC patients	[48]
2123 HBV-positive non-cirrhotic patients and 414 HBV-positive cirrhotic patients, all HEV IgG-negative at baseline	Taiwan	2020	HBeAg-negative chronic HBV patients have an increased long-term risk of cirrhosis, HCC and liver-related death when superinfected with HEV, but not the heterogenous overall cohort	[49]
85 HEV-infected recipients of solid-organ transplants	France	2011	56 patients, all chronically HEV infected transplant recipients; describes HEV as the causative agent of death due to decompensated cirrhosis, and also reports a liver-transplant recipient coinfected with HBV with a relapse of HCC	[50]
238 HIV-infected patients	Spain	2012	Higher seroprevalence of HEV was found in HIV patients with cirrhosis (23%) vs. patients without cirrhosis (6%); liver cirrhosis was the only factor independently associated with the presence of anti-HEV antibodies	[51]
200 HIV-positive individuals	Nepal	2018	HEV IgG-positive HIV-infected patients have a higher Fib-4 score (8.02) compared to HEV IgG-negative HIV-infected patients (1.17)	[52]
Case report, 68-year old male	France	2018	Patient chronically infected with HEV for eight years before the discovery of HCC, where other etiologies for cirrhosis and HCC were ruled out; first case report of HCC most likely as a consequence of cirrhosis due to chronic infection with HEV	[7]
